# PyRAT: An Open-Source Python Library for Animal Behavior Analysis

**DOI:** 10.3389/fnins.2022.779106

**Published:** 2022-05-09

**Authors:** Tulio Fernandes De Almeida, Bruno Guedes Spinelli, Ramón Hypolito Lima, Maria Carolina Gonzalez, Abner Cardoso Rodrigues

**Affiliations:** Post Graduation Program in Neuroengineering, Santos Dumont Institute, Edmond and Lily Safra International Institute of Neuroscience, Macaíba, Brazil

**Keywords:** deep learning, unsupervised learning, behavioral analysis, animal tracking, electrophysiology, neuroscience method

## Abstract

Here we developed an open-source Python-based library called Python rodent Analysis and Tracking (PyRAT). Our library analyzes tracking data to classify distinct behaviors, estimate traveled distance, speed and area occupancy. To classify and cluster behaviors, we used two unsupervised algorithms: hierarchical agglomerative clustering and t-distributed stochastic neighbor embedding (t-SNE). Finally, we built algorithms that associate the detected behaviors with synchronized neural data and facilitate the visualization of this association in the pixel space. PyRAT is fully available on GitHub: https://github.com/pyratlib/pyrat.

## 1. Introduction

Deep learning (DL) and computer vision research fields are improving the performance of image, video and audio data processing (Krizhevsky et al., 2012). The use of these approaches to estimate human and animal pose is increasing rapidly. This new direction stems from several factors, including improved feature extraction, high scalability to data, availability of low-cost hardware designed for DL, and pre-trained models ready for deployment (Toshev and Szegedy, 2014; Redmon et al., 2016; Ilg et al., 2017; Levine et al., 2018; Nath et al., 2019).

Evaluation of animal behavior by human assessment is commonly subjected to inter-rater variability and requires several hours of manual video data evaluation (Spink et al., 2001). Commercial automation software for animal behavior assessment is expensive and rarely provides complex behavioral information. This software uses classical approaches of image processing to track animals' position using contrast or shape data, but they are less reliable to extract detailed information from images (Geuther et al., 2019). In contrast, DL models identify patterns in image data allowing to track the complex movement of specific body parts. Also, DL models allow 3D reconstruction of subjects using single or multiple camera setups instead of complex body markers or light sources to track positions (Nath et al., 2019; Nourizonoz et al., 2020; Dunn et al., 2021).

In the last decade, the scientific community has been incorporating DL algorithms to analyze complex behavior (Gris et al., 2017; Mathis et al., 2018; Jin et al., 2020). Usually, tracking body parts is the first step to classify and/or predict animal behavior. There are several open-source software based on DL to extract body coordinates from videos. However, they only provide the coordinate position for body parts and researchers must implement routines to infer these metrics.

Here, we present a toolbox called Python in Rodent Analysis and Tracking (PyRAT), which is a Python library capable of performing the most common analysis of animal behavior from tracking data. Our user-friendly library can integrate neural data with kinematic metrics, such as velocity, acceleration, presence in areas, and object exploration. We also implemented an unsupervised algorithm to identify and cluster distinct animal behaviors. PyRAT is available in a public repository and can be found at: https://github.com/pyratlib/pyrat.

We believe PyRAT is a useful tool because it can be easily employed to infer some of the most common video analysis metrics through a collection of Python scripts. We developed the library to address real use cases of video analysis, frequently performed in the behavioral field. The outputs of our functions are designed to produce graphics and tables, allowing the selection of subjects and/or time window in each experiment or trial to compare groups. Other open-source libraries presents similar features, however, the behavioral community can benefit from PyRAT simpler and direct approach. We documented the library features with Jupyter notebooks in our repository to guide users to apply our code to their data.

## 2. Materials and Methods

### 2.1. Data

To develop the PyRAT, we used datasets from the Edmond and Lily Safra International Institute of Neuroscience. Adult male Wistar rats (*n* = 12) were placed in an open field arena (59x59 cm with 45 cm tall walls) for 20 min per day for 3 consecutive days. Twenty-four hours later, animals were exposed to two identical objects presented in the open field arena for 5 min. We analyzed 48 videos recorded from a top-down view perspective with a Microsoft LifeCam camera at a resolution of 640 x 480 pixels at 30 frames per second (FPS). Alongside these experiments, neural data from the dorsal hippocampus were collected. All procedures were in accordance with the National Institutes of Health Guide for the Care and Use of Laboratory Animals and approved by a local Animal Care and Use Committee.

Furthermore, we used datasets provided by Sturman et al. (2020) and Fujisawa et al. (2008, 2015) to develop and test PyRAT functions in different scenarios. Sturman et al. (2020) used DeepLabCut to extract poses from mice in an elevated plus maze and an open field arena and provided the videos and the tracking data. Fujisawa et al. (2008) recorded single unit activity in rats performing a working memory task. The dataset is composed of extracellular recordings from the medial prefrontal cortex (64 channels) and dorsal CA1 (a subdivision of the hippocampus, 32 channels) in three rats.

### 2.2. Video Analysis

**Figure 1:**
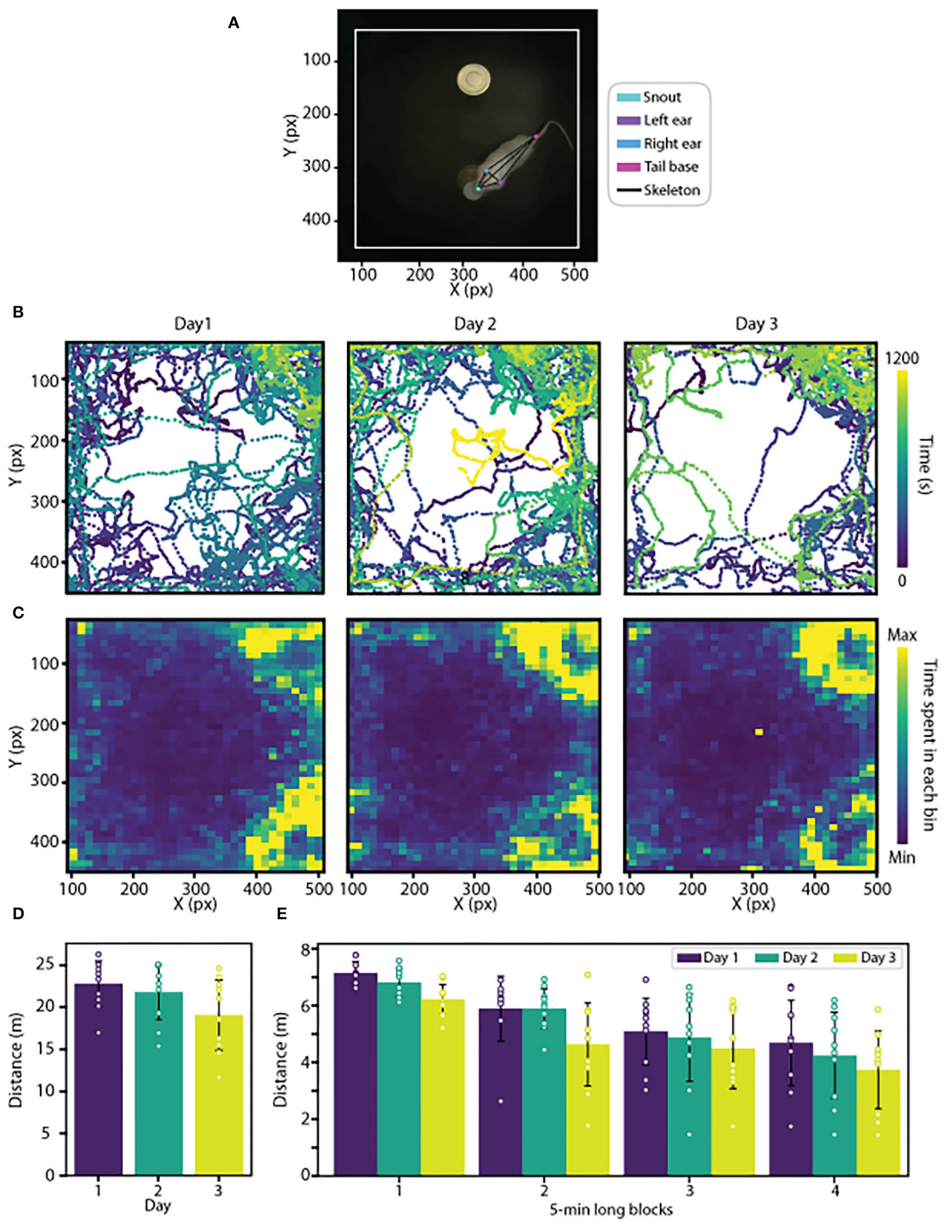
**(A)** Representative image showing the marks of body parts used to train the network and the rat skeleton generated based on these marks. **(B)** Representative trajectory plots of a rat during the exploration sessions of an open field arena carried out on 3 consecutive days. Color variation indicates the moment in time at the rat's location. **(C)** Heatmaps of average trajectories during each exploration session. **(D)** Average distance traveled during each exploration session. **(E)** Average distance traveled during each exploration session is shown in blocks of 5 min per day. Data are expressed as mean ± SD.

For body part tracking, we used DeepLabCut (DLC, version 2.2rc3) (Mathis et al., 2018; Nath et al., 2019). Specifically, we labeled 200 frames (Figure 1A) taken from 5 videos for each scenario (then 95% was used for training). We used a ResNet-50 neural network (Insafutdinov et al., 2016) with default parameters for 3,20,000 training iterations. We validated with 1 number of shuffles and found the test error was: 4.32 pixels, train: 2.69 pixels (image size was 640 by 480). We then used a p-cutoff of 0.9 to condition the X, Y coordinates for future analysis. This network was then used to analyze videos from similar experimental settings.

### 2.3. Library Design and Implementation

Our library is designed to receive as input the DLC tracking data. However, the functions work on pixel space and then can receive any tracking data after applying a few adjustments such as removing the file header, if present and renaming the columns. We developed an example using tracking data from Plexon - available on GitHUb. PyRAT was implemented using Python 3 and the following libraries: NumPy, pandas, scikit-learn, and matplotlib, and hosted in Anaconda and Python Package Index (PyPi).

### 2.4. Unsupervised Behavior Classification

A common task in animal behavior analysis is the identification of distinct behaviors, such as rearing, grooming, nesting, immobility, and left and right turns. To automatically classify behaviors, we used a combination of two unsupervised approaches on each video frame. We used the hierarchical agglomerative clustering algorithm to label the clusters (Lukasová, 1979) and a non-linear technique for dimensionality reduction called t-distributed stochastic neighbor embedding (t-SNE) to visualize the result (Van der Maaten and Hinton, 2008). The input of both algorithms is the distances between labeled body parts. This approach was chosen because the relative distance between body parts is invariant to the animal position in the pixel space. Combining these techniques, we created a map where the distances between the body parts of each frame are transformed into 2D space using t-SNE and the color of each point is determined by the label from hierarchical agglomerative clustering (**Figure 3A**).

To enhance cluster visualization, we optimize the t-SNE hyperparameters according to the heuristics reported in Kobak and Berens (2019). Their approach is based on three steps, (1) the use of Principal Component Analysis (PCA) in t-SNE initialization to preserve the data structure in lower dimensions; (2) set the learning rate as η = *n*/12, where *n* is the number of data points (frames); and (3) set the perplexity hyperparameter, which controls the similarity between points and governs their attraction, as *n*/100. In addition, we implemented three metrics to quantify the quality of the t-SNE output (Kobak and Berens, 2019), (1) the KNN (k-nearest neighbors), which quantifies the preservation of the local structure; (2) the KNC (k-nearest class), which quantifies the preservation of the mesoscale structure; and (3) the CPD (Spearman correlation between pairwise distances), which quantifies the preservation of the global structure.

Since the hyperparameters are not optimized by the learning algorithm, they must be defined *a priori* and selected by trial and error or searching approaches. However, it must be noted that these heuristics have been proven to be useful in empirical tests (Kobak and Berens, 2019).

## 3. Results

### 3.1. Library Features

Python in Rodent Analysis and Tracking is a Python toolbox for the analysis of animal tracking data that is easily accessible by new programmers, entirely developed in Python due to its popularity in the scientific community. The only prerequisite for using our toolbox is having minimal to moderate skills in Python and pandas library. We implemented the functions in a procedural approach instead of using the object-oriented features from Python as we believe that the procedural approach is more user friendly to non-programmers. Moreover, each function encapsulates an analysis, returning all inferred information and graphics. As we employed well-known Python libraries such as pandas, PyRAT can be used with other Python data science libraries such as scipy, sklearn, seaborn, matplotlib, and others.

Python in Rodent Analysis and Tracking functions receive as input a pandas DataFrame with cartesian coordinates of labeled body parts to plot the graphics (data example available on GitHub). The input format is based on the DLC output, which consists of two columns in pixel space (x and y) for each tracked body part. However, any coordinate data organized in DataFrame format can be loaded in PyRAT if it follows the structure of x and y columns for each body part.

To visualize the animal trajectory, we developed two functions. The function Trajectory() plots the body part coordinates across time using a matplotlib colormap (Figure 1B). Here, we use a scatter plot of x and y points and add a third dimension to represent time to facilitate trajectory dynamics. The other function, Heatmap()), generates a heatmap of the animal occupancy in the arena (Figure 1C). The occupancy plot is a 2D histogram that shows the body part occurrence in each spatial bin. We also evaluated the functions in a public dataset of mice performing the open field and the elevated plus maze tasks (Sturman et al., 2020).

**Figure 2:**
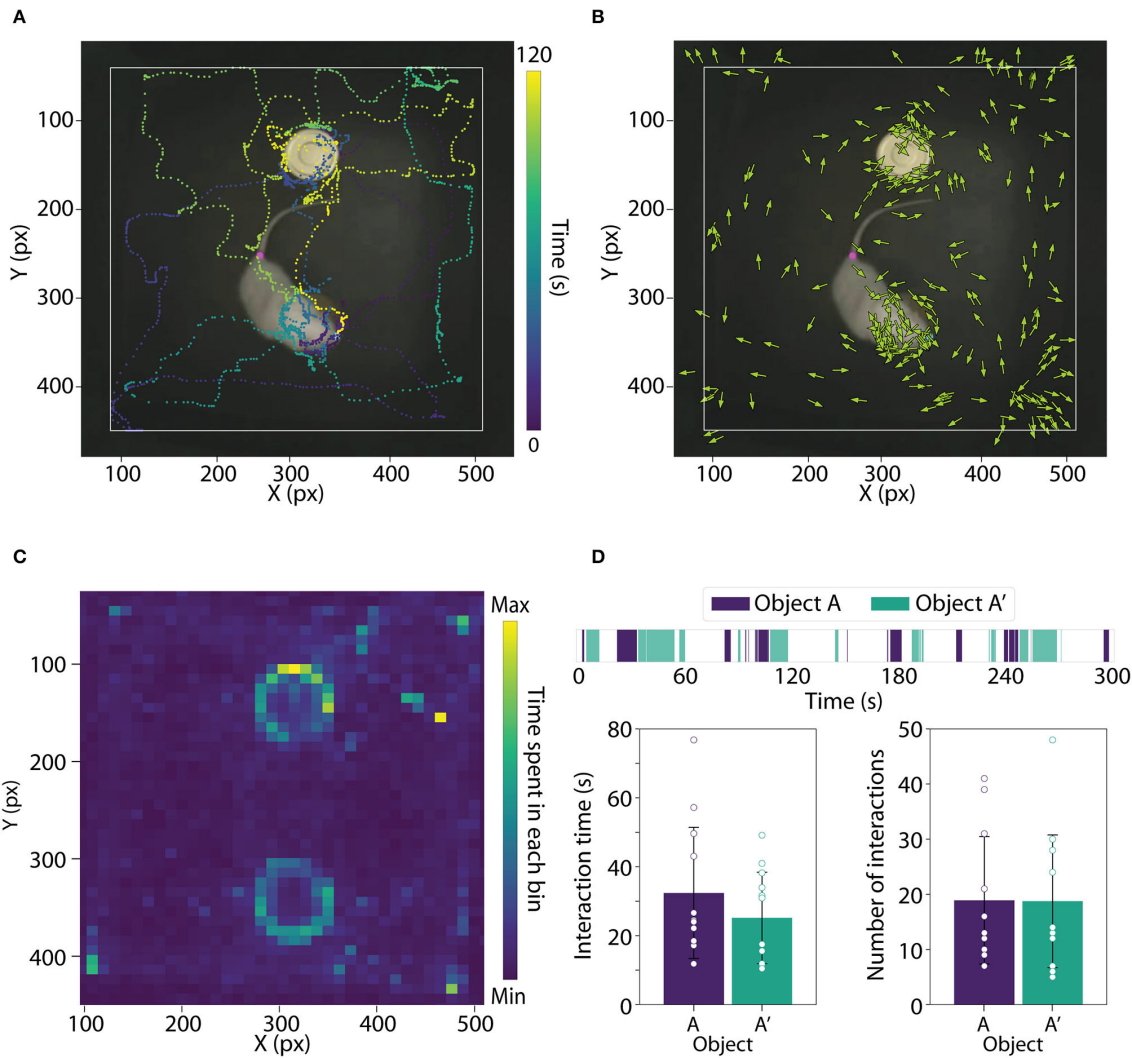
**(A)** Image showing the trajectory of one rat for 120 s based on the snout coordinates. **(B)** Image showing rat body orientation during the entire object exploration session. **(C)** Average heatmap during the entire object exploration session. **(D)** Top: Object interaction across the entire object exploration session; Bottom left: Bar plot showing interaction time with objects A and A'; Bottom right: Bar plot showing the number of interactions with object A and A'. Data are expressed as mean ± SD.

To perform quantitative analyses, we developed the function MotionMetrics(), which estimates speed, acceleration, and traveled distance for each animal (Figure 1D and Supplementary Material). To estimate these metrics, we transform the data from the pixel space to the centimeters space, using a known physical reference, applying the function pixel2centimeters(). Also, the user can define a time interval as an input parameter to calculate the metrics (Figure 1E) and plot trajectory (Figure 2A). To test the accuracy of PyRAT functions, we used a public dataset previously analyzed with EthoVision software (Sturman et al., 2020), and we found equivalent results (data available on PyRAT's GitHub).

Experimental designs that access pathological states or drug effects can use PyRAT to extract head orientation and locomotor activity to compare treatment or conditions (Gulley et al., 2003; Aonuma et al., 2020). The function HeadOrientation() returns head position and orientation in each frame using two points to calculate the element-wise arc tangent between them. The head orientation must be estimated using the neck and snout; however, the same function can estimate body orientation as shown in Figure 2B, using the tail base and snout.

To represent the pattern of object interaction among animal groups, the Heatmap() function can also be used to plot concatenated data, facilitating visual comparison between days, groups, or trials (Figure 2C).

In addition, we developed the FieldDetermination() and Interaction() functions to evaluate the interaction of the animal with defined areas in the pixel space. For this feature, the user must first use the function FieldDetermination() to create circular or a rectangular area. Once the bounding areas are determined, the user must call the function Interaction(), which estimates animal interaction with the areas and returns a DataFrame that reports the beginning and end of each interaction in chronological order. To visualize these outputs, we developed the function PlotInteraction() (Figure 2D).

To summarize data from several subjects and facilitate visualization of behavioral metrics, we included the function Reports(), which combines MotionMetrics() and PlotInteraction() and creates a unified report. The input of this function is a list of the tracking data from each animal and the output is a single DataFrame (examples in Supplementary Material).

**Figure 3:**
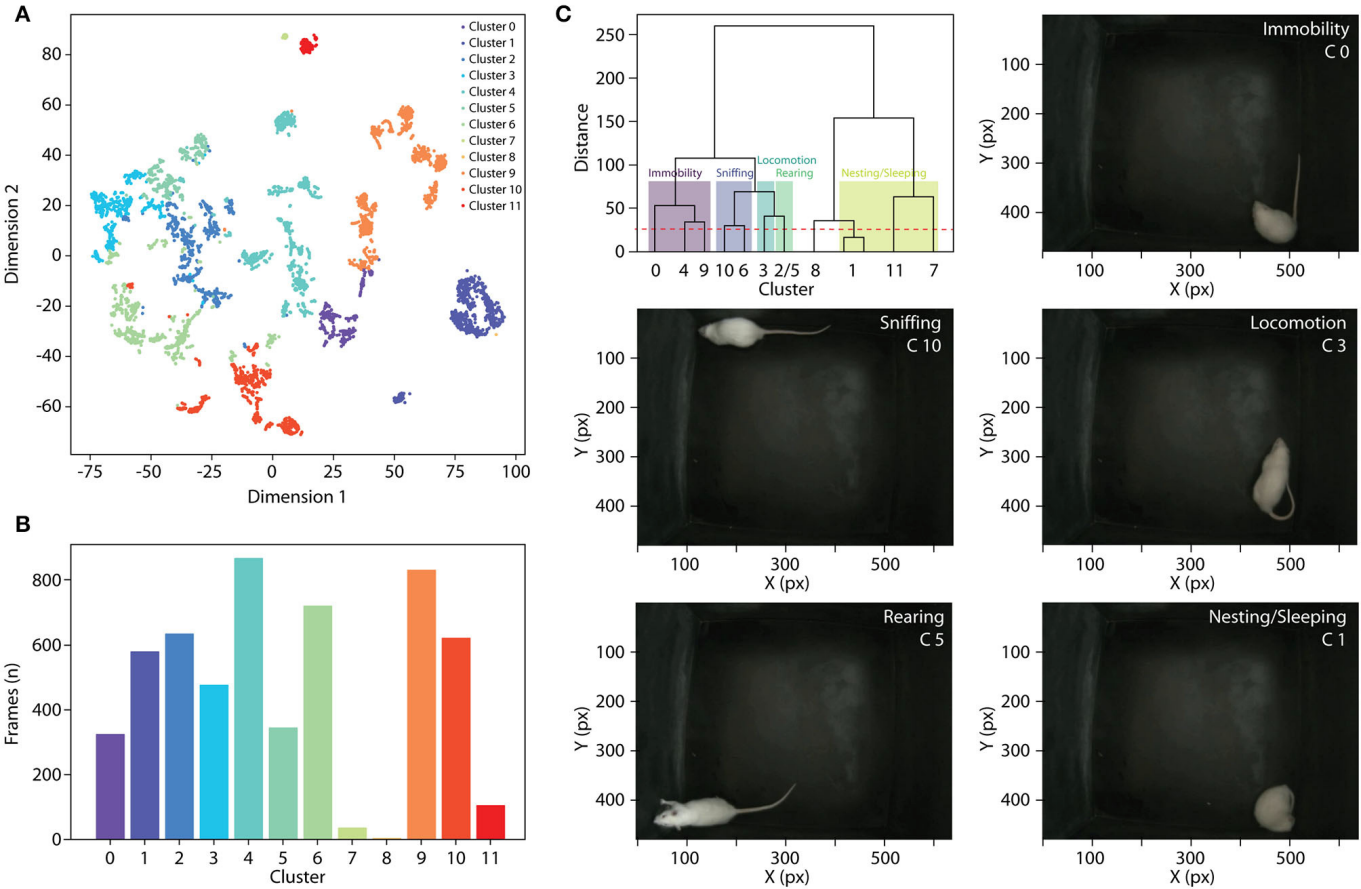
**(A)** Bidimensional projection representing each cluster found by the unsupervised algorithm of behavior classification. **(B)** Histogram showing the number of frames in each cluster. **(C)** Top left: Dendrogram presenting the proximity of the clusters. Clusters with similar behaviors were grouped after visual inspection. We identified five behavioral clusters: immobility, sniffing, locomotion, rearing, and nesting/sleeping. The other images are representative frames showing some of the behavioral clusters identified.

The function ClassifyBehavior() was developed to identify and classify different behaviors. We test this function in two different animal models in the open field task. In rats, 12 clusters were found automatically. The function returns a 2-dimensional color map, a histogram, and a dendrogram to better visualize the results (Figure 3). In addition, the histogram helps to detect mislabeled behaviors considering the number of frames in a cluster. For example, Clusters 7 and 8 presented a small number of frames, and after visual inspection, we confirmed that they were miss-classified samples (Figure 3B). Then, an experienced researcher must inspect the clusters to determine the type of behavior. The dendrogram shows the proximity between clusters and helps to identify the ramifications that represent a class of behavior (Figure 3C). In mice, 5 behavioral clusters were identified (locomotion, left/right turns, sniffing, rearing, and exploration), suggesting that PyRAT is easily generalizable to different experimental setups (data available on PyRAT's GitHub).

**Figure 4:**
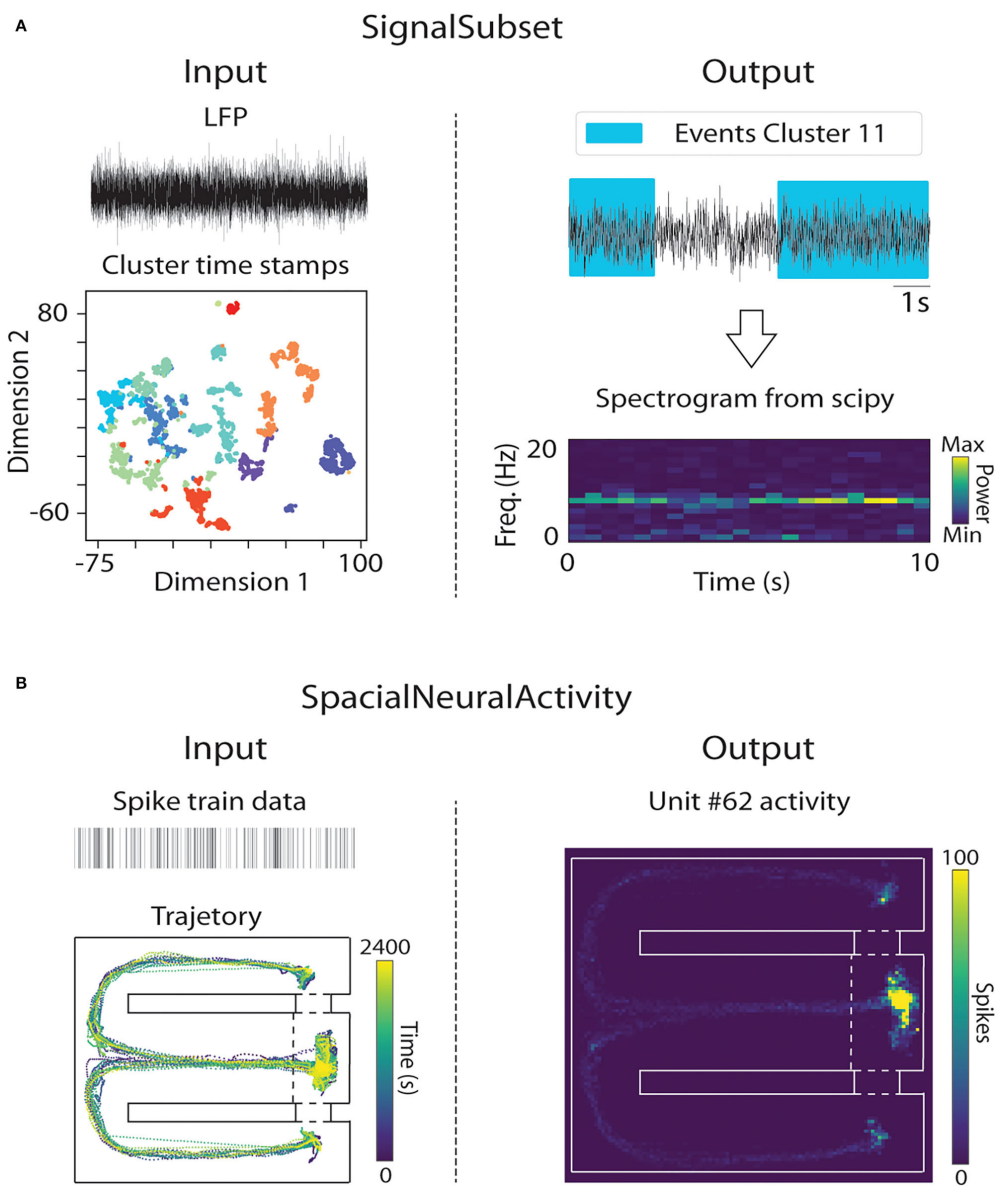
**(A)** Overview of SignalSubset function. Left column: The SignalSubset function receives as input neural data (e.g., raw LFP) and the clustermap produced by ClassifyBehavior. Right column: SignalSubset function returns a list of extracted neural data corresponding to time windows of a determined behavior (e.g., Cluster 11). We also show a representative spectrogram of extracted data. **(B)** Overview of SpatialNeuralActivity function. Left column: The SpatialNeuralActivity function receives as input the neural data (e.g., single unit spike rasterplot) to be shown in pixel space, and the tracking as spatial data. Right column: The SpatialNeuralActivity function returns the quantification of neural activity (spike firing) in each part of the pixel space.

We developed a function to facilitate coupling the tracking data with the analysis of neural signals, in this way, we implemented the SignalSubset() function to extract time windows of defined events based on the interactions (function Interaction() output), the behavioral clusters or even from a list of timestamps (Figure 4A).

The function SpatialNeuralActivity can be used to create a map associating a neural activity to the pixel space. The input of this function is a Dataframe with the x and y of each frame together with a third column with the neural activity to be visualized. The output is a 2D NumPy array with the mean activity in each discrete space of the map. We used neural data published in Fujisawa et al. (2008) to develop an example of spike triggered activity for some units in a T-maze (Figure 4B). We are still developing this function to add more features, e.g., to plot the mean band of an LFP channel in the map instead of the spike data. The results and the code are available on PyRAT's GitHub.

### 3.2. User Guide

Python in Rodent Analysis and Tracking is a user-friendly Python toolbox to automate the analysis of animal tracking and neural data. Toolbox functions are documented, and here, we describe how to use the key features. PyRAT can be installed using pip install pyratlib. Then, it is necessary to import the following libraries:


      import pyratlib as rat
      import pandas as pd


Subsequently, the user must read tracking data as a DataFrame, e.g., using the read_csv() function from pandas. This Dataframe will be used as input on the majority of PyRAT functions. Here, we show how to plot the trajectories and the heatmap:


      data = pd.read_csv('your_data_path.csv')
  
      rat.Trajectory(data, bodyPart = 'tail', bodyPartBox = 'tail')
      rat.Heatmap(data, bodyPart = 'tail', bins = 10, vmax = 50)


To plot the trajectory, the user must define a body part in the function Trajectory using the bodyPart parameter which is the column name of the chosen body part. The function Heatmap() uses the bodyPart and the parameters bins and vmax, which determine the resolution and color scale of the plot.

Another PyRAT feature is the quantification of the interaction between a body part and an area. This interaction can be calculated with the function Interaction() and defining a bounding area by passing the size and coordinates of the vertices. The function FieldDetermination() allows the visualization of areas in the pixel space, according to the tracking data. Also, we developed the function PlotInteraction() to plot the beginning, end, and duration of interactions with each bounding area across time:


     obj_dict = {'Obj_1': [1,0,0,0,430, 35,90,75],
                 'Obj_2': [1,0,0,0,430,380,90,75]}
  
     objects = rat.FieldDetermination(posit = obj_dict)
     interactions = rat.Interaction(data,'snout',objects)
     rat.PlotInteraction(interactions)


In the example above, two areas representing objects in distinct positions were passed as input, and the output is a DataFrame with the timestamps of each object interaction. The function PlotInteraction() plots object interactions across time (Figure 2D).

The function ClassifyBehavior() is a behavioral classifier and receives as parameters the tracking DataFrame, the video directory, the selected body parts, and the distance:


      rat.ClassifyBehavior(df,
                           video = 'path',
                           bp_list = ['snout', 'ear_R', 'ear_L', 'tail'],
                           distance = 28)


The distance metric passed in this function is Ward's distance and defines the threshold above which the clusters will not be merged.

To facilitate the analysis of neural signals recorded during behavioral tasks, we developed the function Interaction() to extract timestamps of events of interest and the function SignalSubset() to extract epochs of the neural signal. An example of neural data input is available in Supplementary Material. We used files from Plexon and Blackrock Neurotech, but data from other acquisition systems can be used.


      subsets = rat.SignalSubset(signal, freq = 1000,
                                 fields = interactions)


SignalSubset() returns the extracted data organized in a dictionary with the number of the epoch as the key. In addition, it can extract the time of a selected behavioral cluster. For this, it is necessary to use the cluster output from ClassifyBehavior() as input to the IntervalBehaviors() function, which will return a dictionary with the time windows when each behavior was manifested (documented in GitHub). This function facilitates data processing and allows saving the dictionary, speeding up data loading.

The function Reports(), which summarizes data from several animals, receives as input the lists with DataFrames and the file names, as well as the body part of interest to extract the metrics and, if necessary, an area to calculate interactions:


      list_df = [df01,df02,df03,df04,df05,df06,
                 df07,df08,df09,df10,df11,df12]
      names = ['RAT01','RAT02','RAT03','RAT04','RAT05','RAT06',
               'RAT07','RAT08','RAT09','RAT10','RAT11','RAT12']
      report = rat.Reports(df_list = list_df,list_name = names,
                           bodypart = 'snout',fields = objects)


## 4. Discussion

We presented the PyRAT, a library for animal tracking data analysis developed to be accessible to less experienced programmers. We implemented functions to infer common animal behavioral metrics used in the literature, such as object interaction (duration and number of interactions), traveled distance, speed, and time spent in different areas (Lima et al., 2009; Gonzalez et al., 2019; Rossato et al., 2019; Moura et al., 2020). Also, we implemented functions to infer animal behavior from tracked body parts in each frame using unsupervised approaches. If video recordings are synchronized with neural data, PyRAT can be used to extract epochs based on specific behaviors or metrics. Finally, our results indicate that PyRAT analyzes tracking data from different animal models if videos were acquired from a top-down perspective.

There is similar software that can analyze tracking data as PyRAT, such as Traja, DLCAnalyzer, SimBA, and B-SOiD. Traja is a Python library that can analyze tracking data from coordinate data from any setup but does not infer behavioral metrics. DLCAnalyzer is a collection of R scripts that processes DLC files and quantifies motion metrics and behavior using supervised algorithms (Sturman et al., 2020). Simple Behavioral Analysis (SimBA) is software with an easy-to-use interface that analyzes video or tracking data and applies a pre-trained supervised classifier to cluster behaviors (Nilsson et al., 2020). However, the SimBA interface only works in Windows, limiting its usability on other platforms. B-SOiD is an open-source package that identifies behavior by combining supervised and unsupervised algorithms (Hsu and Yttri, 2021) and works in mice, rats, and humans. B-SOiD analyzes videos acquired from different perspectives, showing the best results from bottom-up recordings. For further discussion and comparison between these tools refer to Panadeiro et al. (2021); von Ziegler et al. (2021). In contrast with other tools, PyRAT can be used in any operational system, does not need pre-trained classifiers, works without a graphic interface, and provides interactive documentation using Jupyter notebooks.

Python in Rodent Analysis and Tracking is easier to use than other alternatives as it is a collection of functions, and the user just needs to input the tracking data to get the results and graphics following the step-by-step tutorial included in the documentation. In addition, PyRAT has a low learning curve, as its implementation is based on procedural programming. We designed the library to display metrics and graphics for all recorded sessions with a few lines of code. It does not have software requirements besides Python and widely used libraries, such as sklearn, pandas, and matplotlib. In summary, we present an open-source Python library to process tracking data, extract behavior and associate this information with neural data in a user-friendly approach.

## Data Availability Statement

The original contributions presented in the study are publicly available. This data can be found here: GitHub: https://github.com/pyratlib/pyrat; Zenodo: https://zenodo.org/record/5883277.

## Ethics Statement

The animal study was reviewed and approved by Animal Research Ethics Committee of Santos Dumont Institute.

## Author Contributions

TD, BS, and AR designed, wrote, tested the library, and performed the analysis of the examples. RH and MG evaluated the algorithms. TD documented the library. TD, RH, MG, and AR wrote the manuscript. All authors contributed to the article and approved the submitted version.

## Funding

“This study was financed in part by the Coordenação de Aperfeiçoamento de Pessoal de Nível” “Superior – Brasil (CAPES) – Finance Code 001.”, Conselho Nacional de Desenvolvimento Científico e Tecnológico (CNPq), Ministério da Educação (MEC), and Instituto Santos Dumont (ISD).

## Conflict of Interest

The authors declare that the research was conducted in the absence of any commercial or financial relationships that could be construed as a potential conflict of interest.

## Publisher's Note

All claims expressed in this article are solely those of the authors and do not necessarily represent those of their affiliated organizations, or those of the publisher, the editors and the reviewers. Any product that may be evaluated in this article, or claim that may be made by its manufacturer, is not guaranteed or endorsed by the publisher.
